# IFN-α suppresses HTLV-1 expression via PKR in infected cells and renders them susceptible to AZT through p53 activation in AZT/IFN-α treatment

**DOI:** 10.1186/1742-4690-11-S1-O24

**Published:** 2014-01-07

**Authors:** Shuichi Kinpara, Mami Kijiyama, Ayako Takamori, Atsuhiko Hasegawa, Amane Sasada, Takao Masuda, Yuetsu Tanaka, Atae Utsunomiya, Mari Kannagi

**Affiliations:** 1 Department of Immunotherapeutics, Graduate School, Tokyo Medical and Dental University, Tokyo, Japan; 2 Department of Immunology, Graduate School of Medicine, University of the Ryukyus, Okinawa, Japan; 3 Department of Hematology, Imamura Bun-in Hospital, Kagoshima, Japan

## 

HTLV-1 expression is maintained at low levels in vivo through unknown mechanisms. Interferon-α (IFN-α) has been used in combination with zidovudin (AZT) to treat adult T-cell leukemia/lymphoma (ATL) patients with favorable effects, although the mechanism is also unclear. We previously reported that HTLV-1 gene expression could be markedly suppressed by stromal cells through a type-I IFN response in IL-2-dependent HTLV-1-infected T-cells (ILTs) derived from ATL patients. Here, we found that treatment with IFN-α alone produced suppressive effects on HTLV-1 gene expression in ILTs and also on the spontaneous HTLV-1 induction in short-term cultured primary ATL cells. Following IFN-α treatment, the levels of intracellular Tax protein decreased earlier than those of HTLV-1 mRNA in ILTs. An RNA-dependent protein kinase (PKR) inhibitor reversed IFN-α-mediated suppression of Tax in ILTs, suggesting the involvement of a post-transcriptional mechanism mediated by PKR in the suppression. IFN-α also induced cell cycle arrest at the G0/G1 phase in ILTs. We further found that AZT combined with IFN-α induced cell apoptosis in ILTs, associated with phosphorylation of p53 and enhanced expression of genes responsive to p53, whereas AZT alone did not affect cell viability or viral expression. Our results suggest that ILTs and ATL cells retain susceptibility to type-I IFNs, which suppress HTLV-1 gene expression primarily at a post-transcriptional level and potentially allow for signaling through the p53 pathway in combination with AZT. These findings would partly explain how HTLV-1 gene expression is regulated in vivo, and how AZT/IFN-α produces therapeutic effects in ATL.

